# Supplementary material for: Biomolecular Adsorprion at ZnS Nanomaterials: A Molecular Dynamics Simulation Study of the Adsorption Preferences, Effects of the Surface Curvature and Coating
Source: Nanomaterials (Basel). 2023 Aug 2;13(15):2239. doi: 10.3390/nano13152239 (PMC10421200; doi:10.3390/nano13152239)
Supplement: Supplementary file 1 [file nanomaterials-13-02239-s001.zip › nanomaterials-2508292-supplementary.pdf]

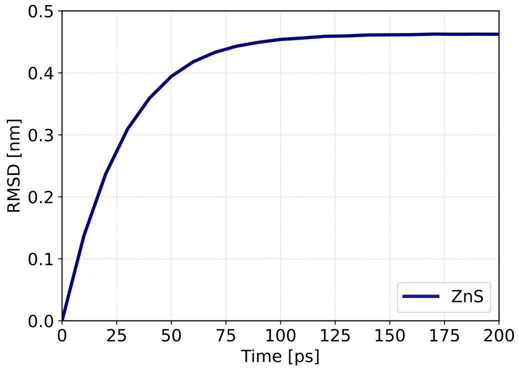

Figure S1. Root mean square deviation analysis for ZnS crystal structure.

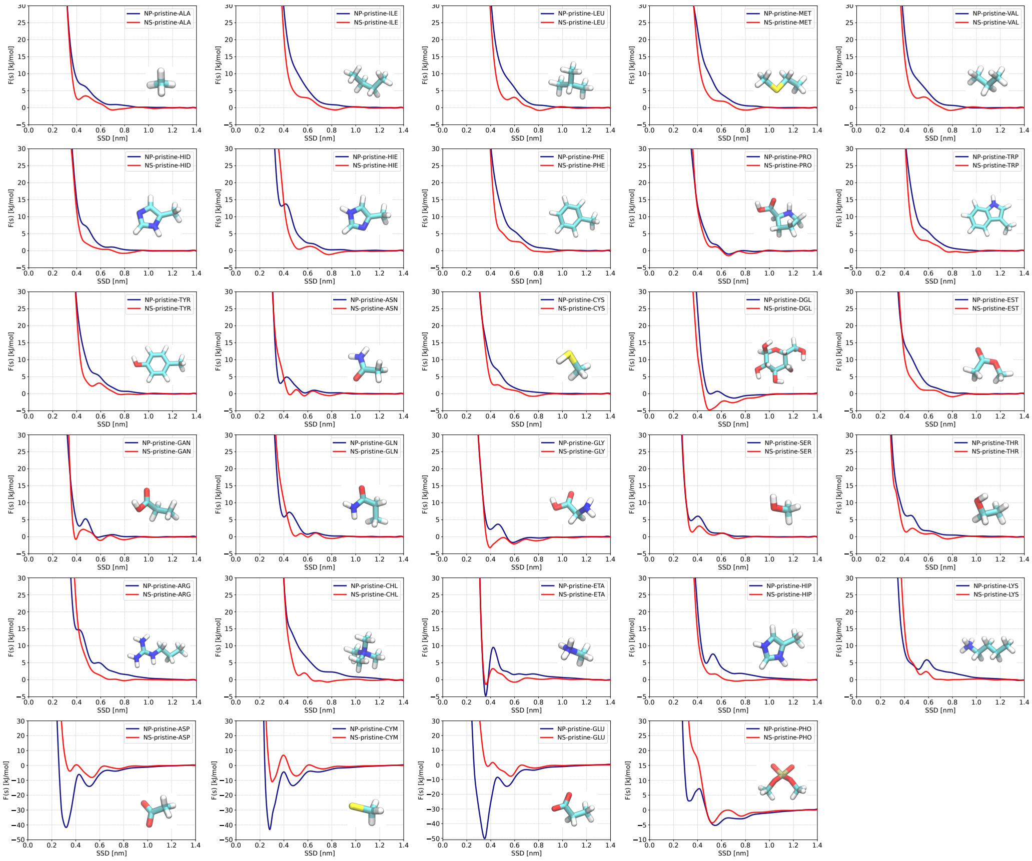

Figure S2. Adsorption free energy profiles as functions of the surface separation distance (SSD) for different biomolecules on ZnS (110) plane and ZnS nanoparticle in pristine (non-coated) form. The red curves correspond to ZnS (110) nanoslabs (NS), and the blue curves correspond to ZnS nanoparticle (NP). The chemical structure of biomolecules are shown in each corresponding plot.

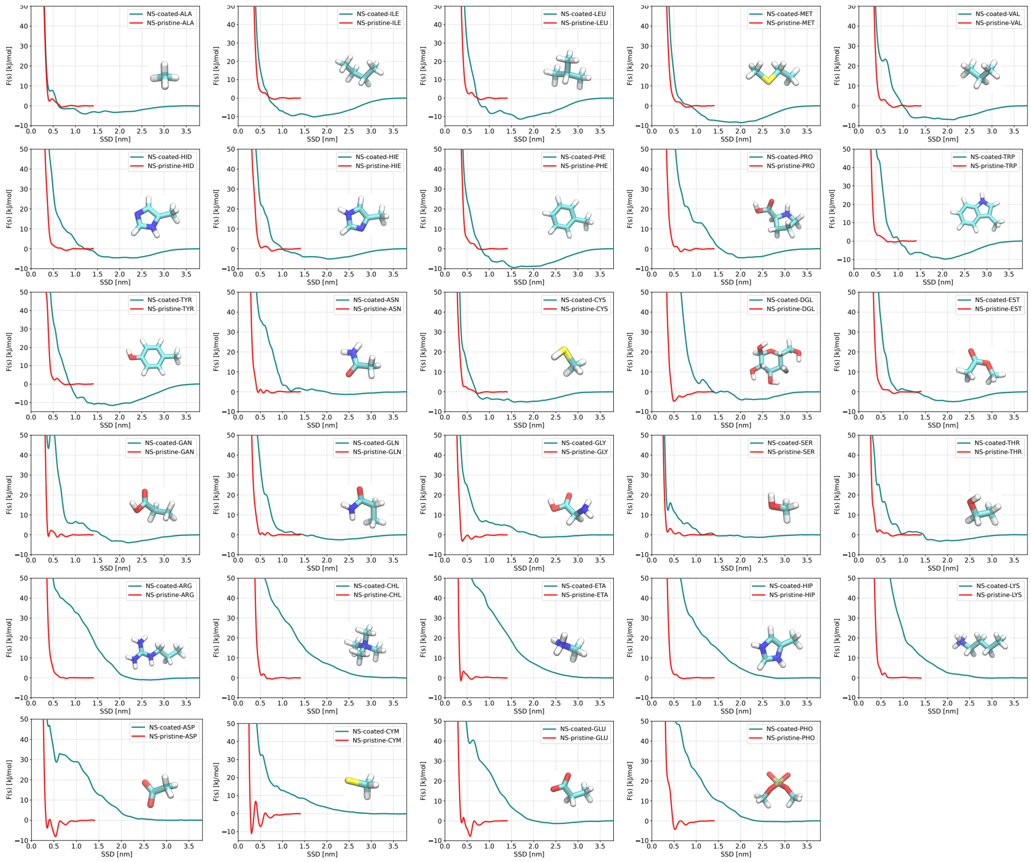

Figure S3. Adsorption free energy profiles as functions of the surface separation distance (SSD) for different biomolecules on ZnS (110) plane in pristine (non-coated) and coated (with PMMA) forms. The red and green curves correspond to the pristine and coated form of ZnS (110) respectively. The chemical structure of biomolecules are shown in each corresponding plot.

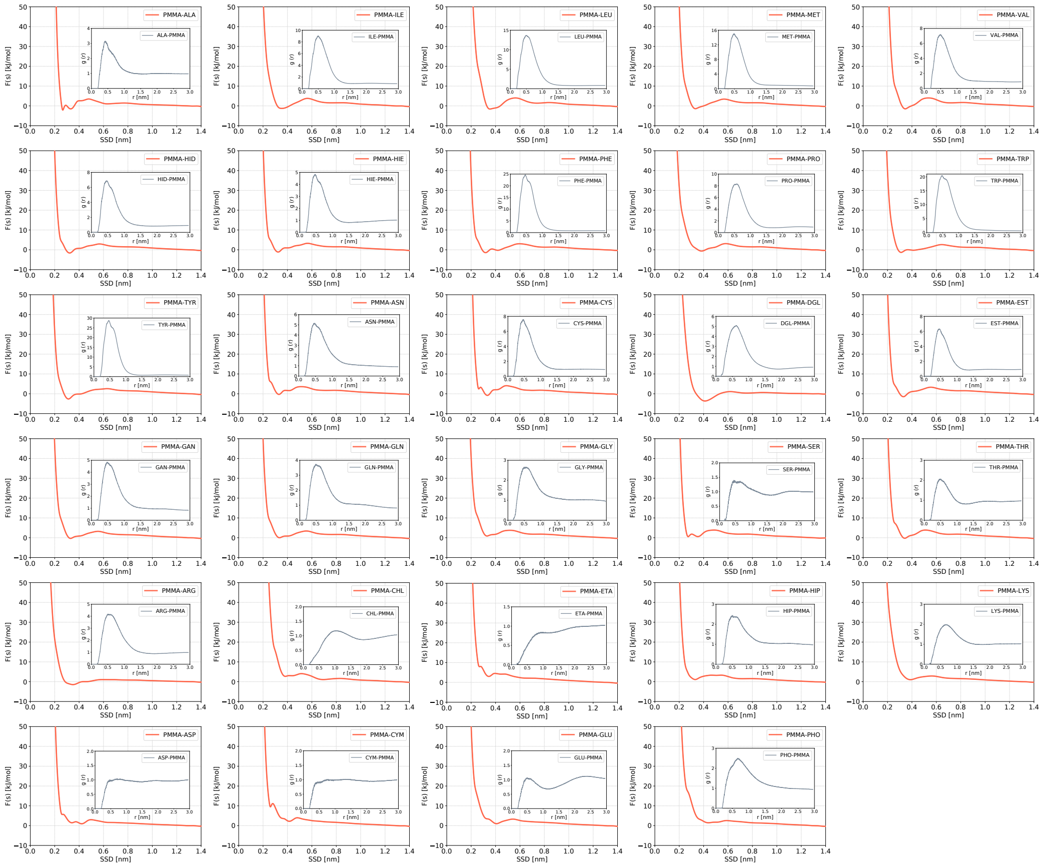

Figure S4. The outer plots represent adsorption free energy profiles as functions of the minimum distance between the center of mass of each biomolecules to the nearest atom of 3-MMA molecule. The RDF of each biomolecule to a 3-MMA molecule are shown in the inner plots.

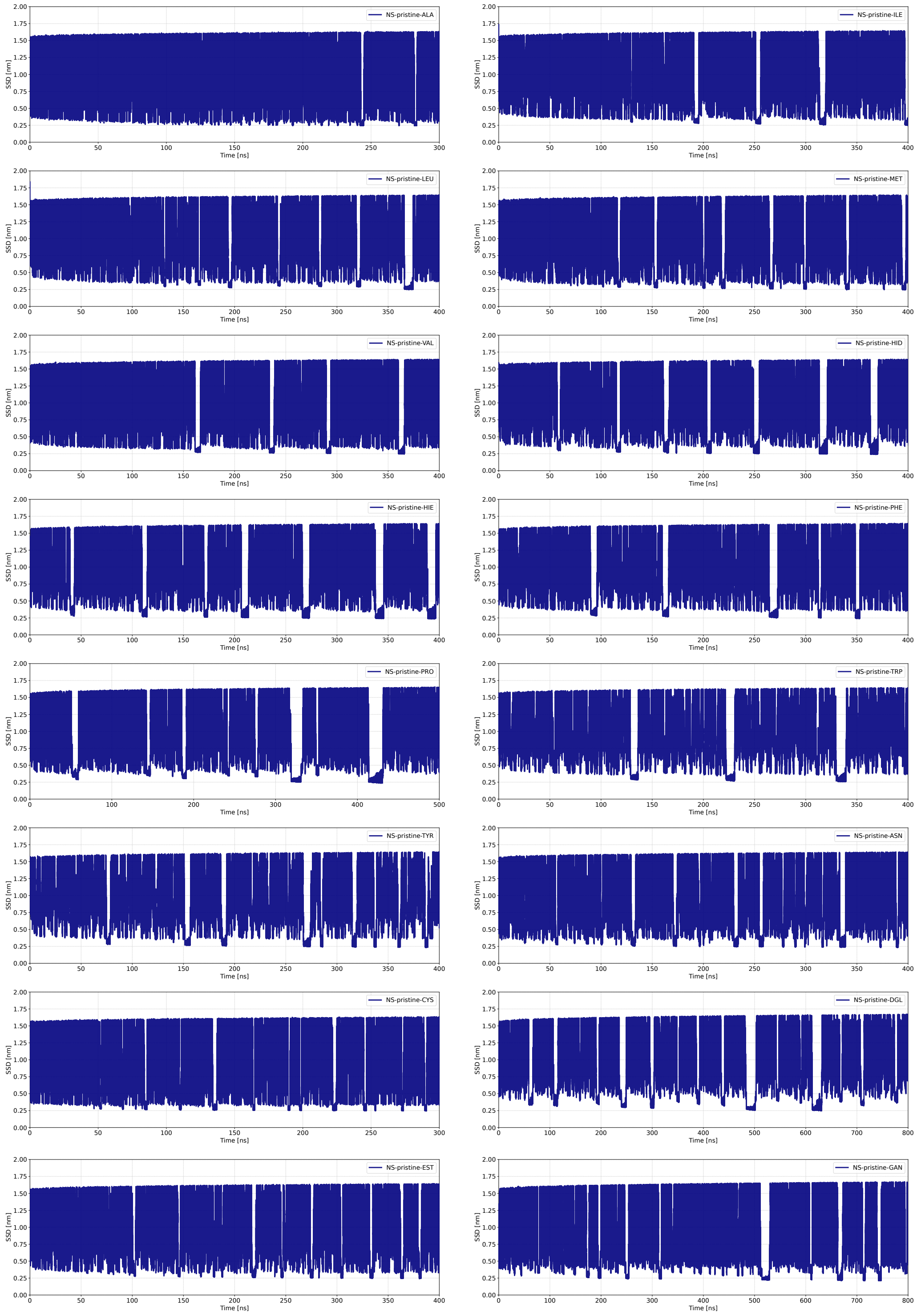
NS-pristine-ALA
NS-pristine-ILE
NS-pristine-LEU
NS-pristine-MET
NS-pristine-VAL
NS-pristine-HID
NS-pristine-HIE
NS-pristine-PHE
NS-pristine-PRO
NS-pristine-TRP
NS-pristine-TYR
NS-pristine-ASN
NS-pristine-CYS
NS-pristine-DGL
NS-pristine-EST
NS-pristine-GAN
SSD [nm]
Time [ns]

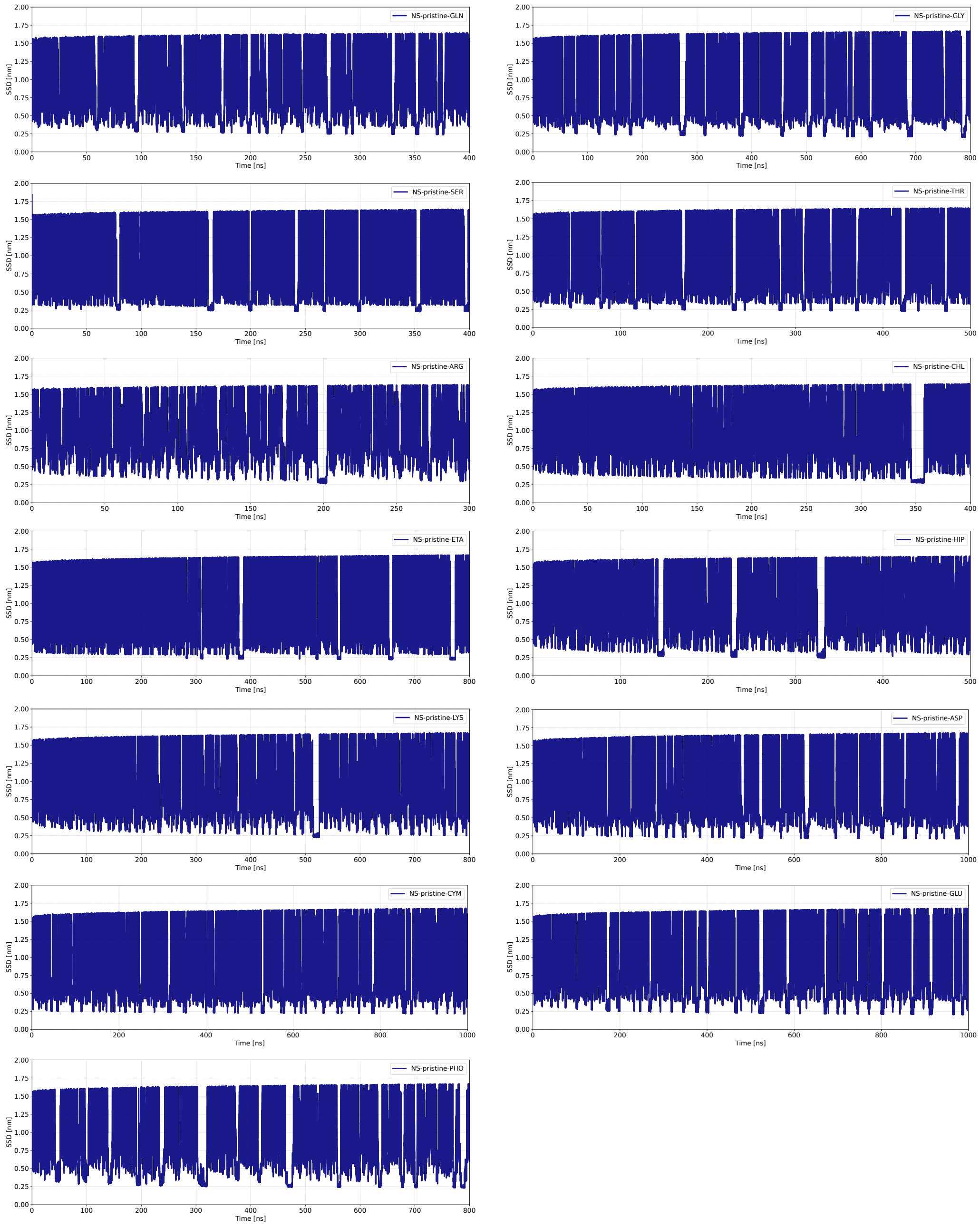

Figure S5. Surface separation distance (SSD) during metadynamics simulation of biomolecules on the pristine ZnS (110) slabs.

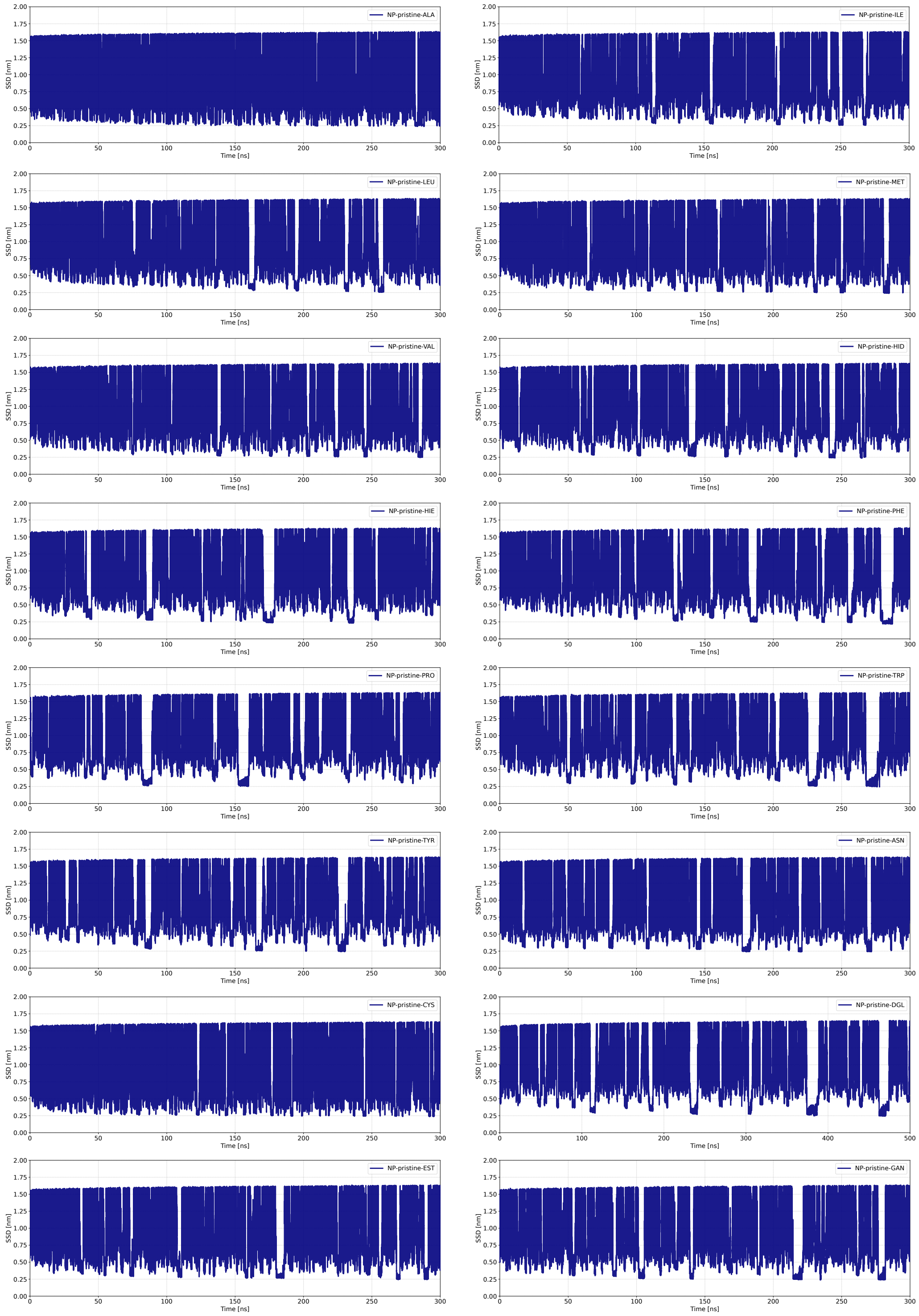

NP-pristine-ALA
NP-pristine-ILE
NP-pristine-LEU
NP-pristine-MET
NP-pristine-VAL
NP-pristine-HID
NP-pristine-HIE
NP-pristine-PHE
NP-pristine-PRO
NP-pristine-TRP
NP-pristine-TYR
NP-pristine-ASN
NP-pristine-CYS
NP-pristine-DGL
NP-pristine-EST
NP-pristine-GAN
SSD [nm]
Time [ns]

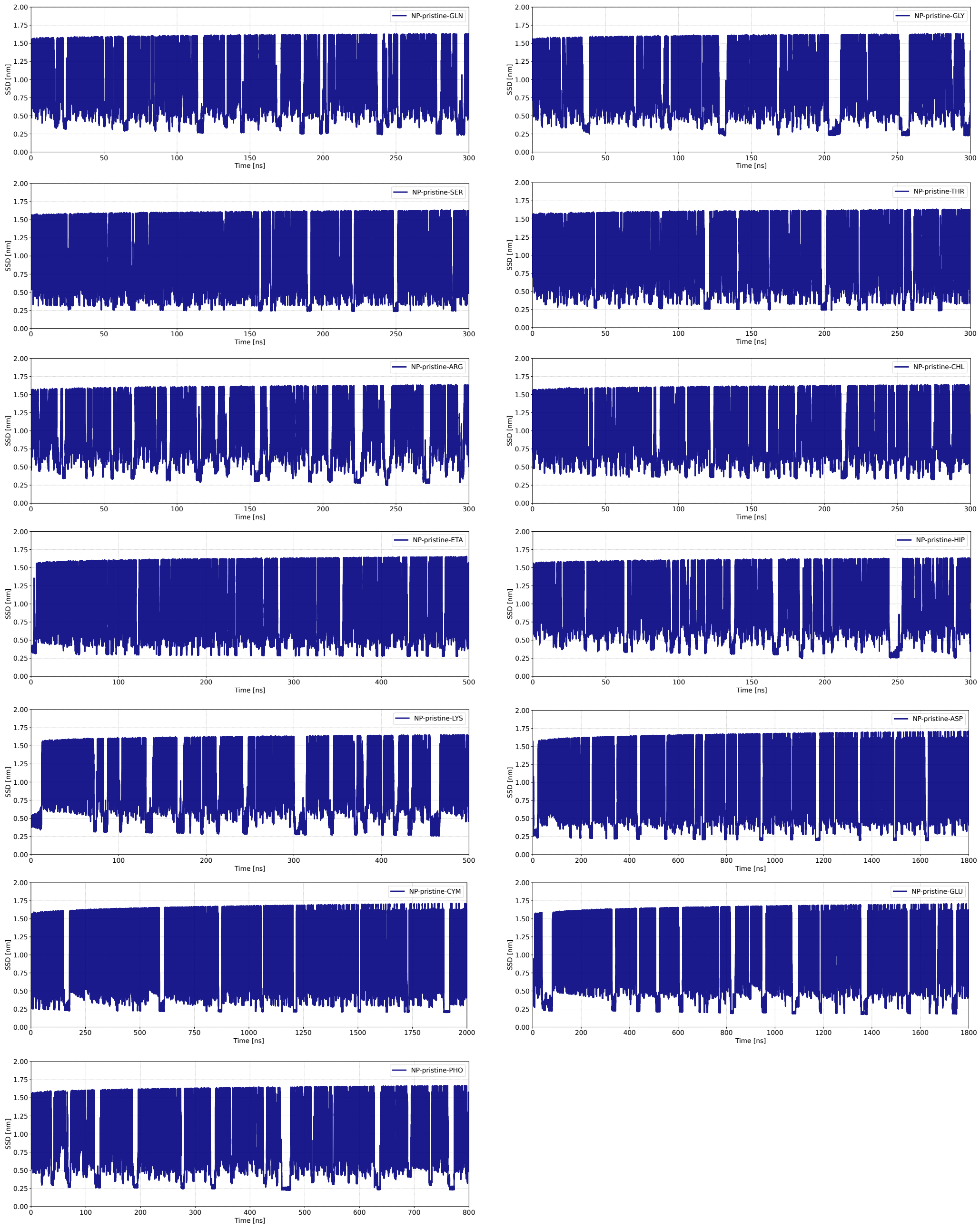

Figure S6. Surface separation distance (SSD) during metadynamics simulation of biomolecules on the pristine ZnS nanoparticle.

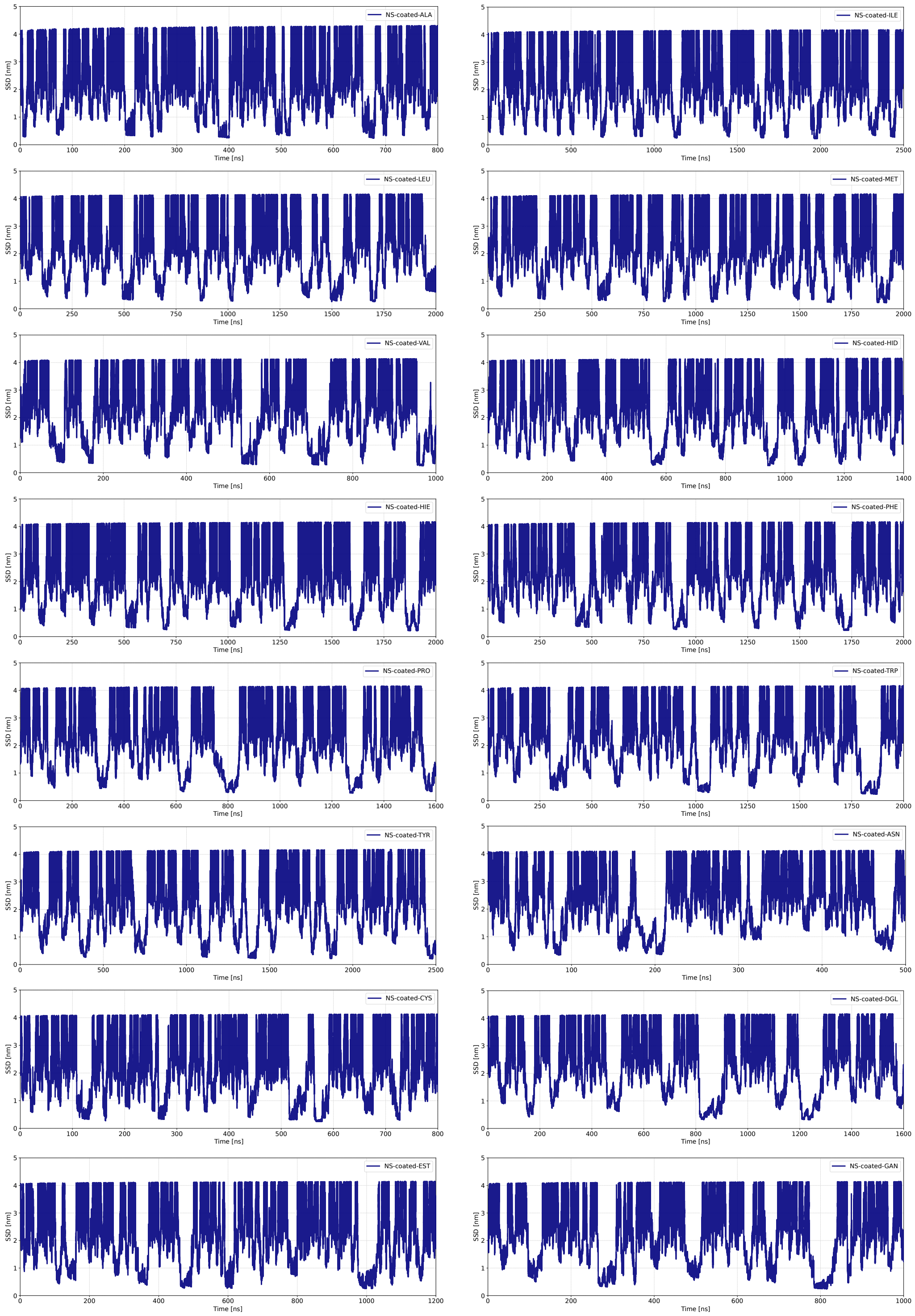
NS-coated-ALA
NS-coated-ILE
NS-coated-LEU
NS-coated-MET
NS-coated-VAL
NS-coated-HID
NS-coated-HIE
NS-coated-PHE
NS-coated-PRO
NS-coated-TRP
NS-coated-TYR
NS-coated-ASN
NS-coated-CYS
NS-coated-DGL
NS-coated-EST
NS-coated-GAN
SSD [nm]
Time [ns]

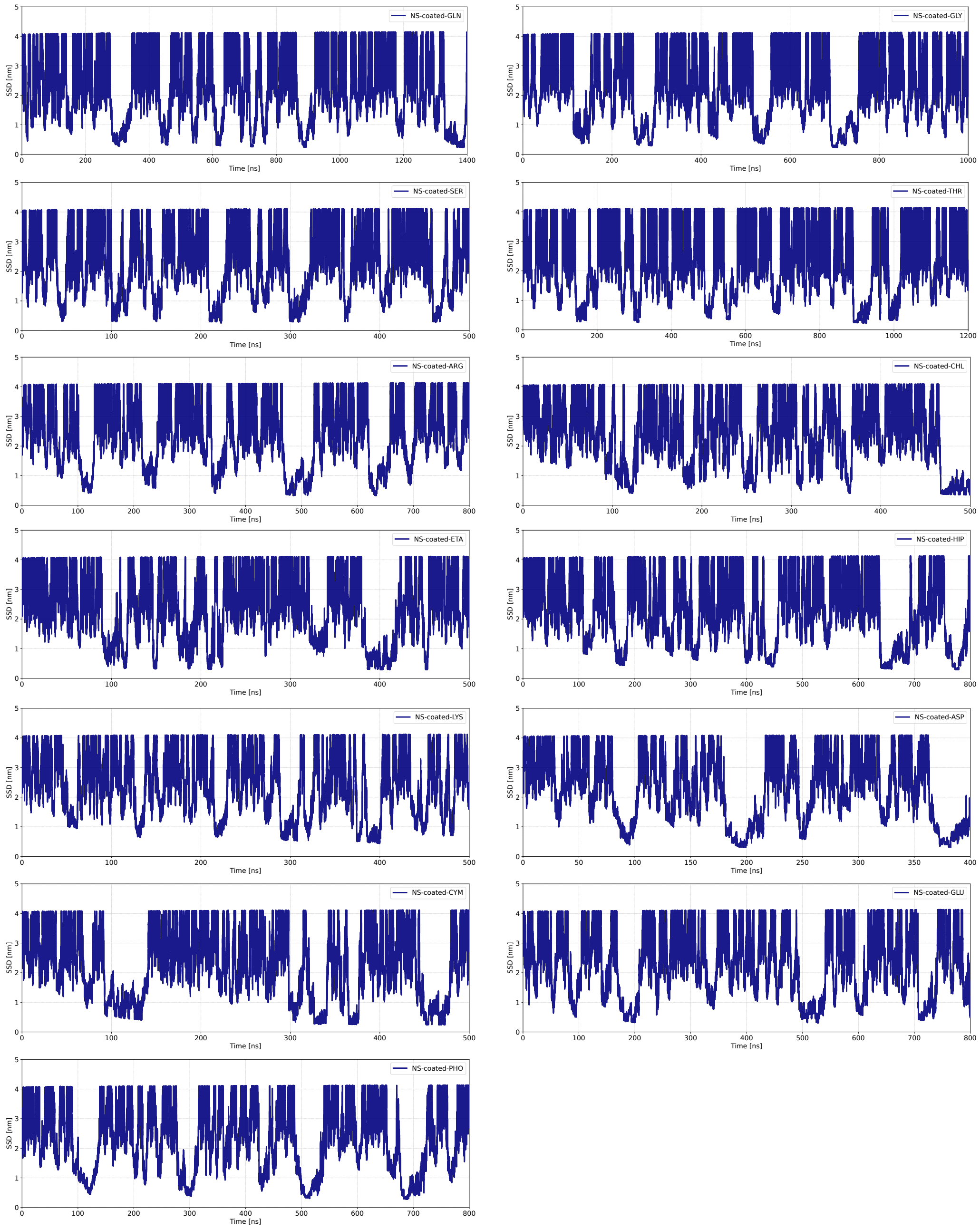

Figure S7. Surface separation distance (SSD) during metadynamics simulation of biomolecules on the coated ZnS (110) slabs.

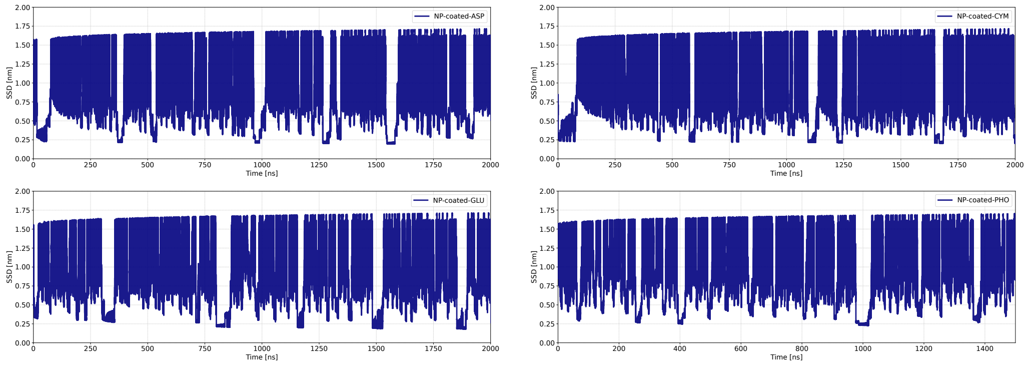

Figure S8. Surface separation distance (SSD) during metadynamics simulation of biomolecules on the coated ZnS nanoparticles.

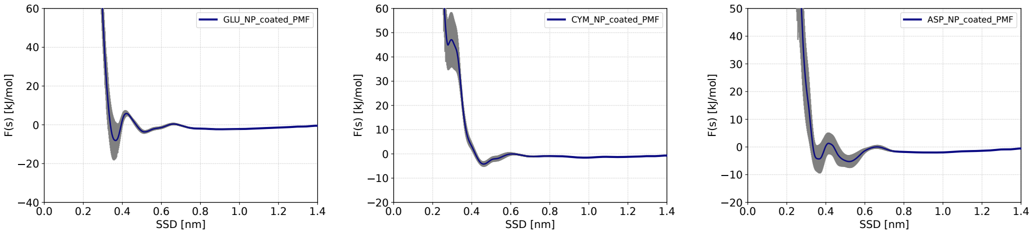

Figure S9. Adsorption profiles of biomolecules at ZnS nanoparticles. The gray curves show errors.
